# Population HIV Viremia and Epidemic Transition: Findings From Sequential Surveys in 6 Sub-Saharan African Countries

**DOI:** 10.1093/ofid/ofag482

**Published:** 2026-08-08

**Authors:** Mansoor Farahani, Suzue Saito, Shannon M Farley, Theodore F Smart, Giles A Reid, Wilford L Kirungi, Owen Mugurungi, Rose Kolola Nyirenda, Wafaa M El-Sadr

**Affiliations:** ICAP at Columbia University, Mailman School of Public Health, New York, New York, USA; ICAP at Columbia University, Mailman School of Public Health, New York, New York, USA; ICAP at Columbia University, Mailman School of Public Health, New York, New York, USA; ICAP at Columbia University, Mailman School of Public Health, New York, New York, USA; ICAP at Columbia University, Mailman School of Public Health, New York, New York, USA; AIDS Control Division, Ministry of Health, Kampala, Uganda; AIDS and TB Unit, Ministry of Health and Child Care, Harare, Zimbabwe; Department of HIV & AIDS, Ministry of Health, Lilongwe, Malawi; ICAP at Columbia University, Mailman School of Public Health, New York, New York, USA; Department of Epidemiology, Columbia University Mailman School of Public Health, New York, New York, USA

**Keywords:** epidemic transition, HIV, incidence-to-prevalence ratio, population viremia, viral load suppression

## Abstract

**Background:**

Testing and treatment targets may not fully capture the progress of the epidemic. Whether cascade gains translate into short-term reductions in new infections, as reflected by the incidence-to-prevalence ratio (IPR), remains uncertain.

**Methods:**

We analyzed 2 rounds of Population-based HIV Impact Assessment surveys in Eswatini, Lesotho, Malawi, Tanzania, Uganda, and Zimbabwe (Round 1, 2015–2017; Round 2, 2019–2023) among adults aged 15–59 years. We estimated human immunodeficiency virus (HIV) prevalence, the 95–95–95 cascade, population viral load suppression (PVLS), population viremia (PV), HIV incidence, and IPR using survey-weighted methods. Population viremia was categorized as undiagnosed and viremic, diagnosed but not on antiretroviral therapy (ART), or on ART but not suppressed.

**Results:**

The sample included 113 970 Round 1 and 112 445 Round 2 participants. Across countries, PVLS ranged from 52.0% to 72.4% in Round 1 and from 74.5% to 88.0% in Round 2; PV ranged from 2.4% to 8.3% in Round 1 and from 1.0% to 4.7% in Round 2. In Round 2, undiagnosed infection accounted for most PV in 4 countries (53.1%–71.4%). Incidence estimates were lower in Round 2 in 5 countries, but CIs were wide and overlapping. The incidence-to-prevalence ratio ranged from 3.0% to 6.7% in Round 1 and from 2.1% to 5.1% in Round 2; estimates were <3% in Eswatini, Lesotho, and Malawi, but CIs included 3%.

**Conclusions:**

Population viral load suppression increased, and PV declined in all countries, but precision for short-term IPR change was limited. Residual viremia was concentrated among undiagnosed adults, supporting targeted testing, rapid linkage, and treatment continuity.

## Teaser

Across 6 sub-Saharan African countries, sequential population surveys showed increased population viral load suppression and decreased population viremia, but incidence trends remained uncertain. Residual viremia was concentrated among undiagnosed people with HIV, supporting targeted testing, linkage, and sustained treatment engagement.

Despite substantial gains in confronting the human immunodeficiency virus (HIV) epidemic, an estimated 1.3 million (1.0–1.7 million) people acquired HIV in 2024 [1]. Progress has been driven largely by the scale-up of HIV testing and antiretroviral therapy (ART), fueling momentum toward the Joint United Nations Programme on HIV/AIDS (UNAIDS) 95–95–95 targets of diagnosis, treatment, and viral load suppression (VLS) and an increase in population VLS [2, 3]. However, cascade metrics alone do not fully capture epidemic dynamics, as complementary epidemiological indicators are needed to track changes in the HIV epidemic, including changes in HIV transmission and mortality over time [4, 5]. The ratio-based metric, the incidence-to-prevalence ratio (IPR), has been proposed as a pragmatic summary of the epidemic trajectory, and a decrease in IPR over time would indicate progress toward an important benchmark in epidemic transitions. While IPR <3% has been proposed as a benchmark for HIV epidemic transition, such thresholds remain contested because ratio metrics are sensitive to demographic change, improved survival, and measurement uncertainty, and they should not be used as stand-alone criteria for epidemic transition [4, 5].

Population viremia (PV), defined here as the proportion of all adults who are HIV-positive with viral loads ≥1000 copies/mL, provides a complementary, programmatically actionable measure that links cascade performance to transmission potential. In a pooled analysis of 4 universal testing and treatment trials in eastern and southern Africa, declines in PV were strongly associated with declines in HIV incidence, supporting PV as a potential marker of epidemic momentum [6]. Identifying where viremia persists across cascade stages may offer specific loci for interventions.

Growing evidence suggests that residual transmission potential is often concentrated among people with HIV (PWH) who remain undiagnosed and among those who disengage from care and experience treatment interruption. Recent research comparing several HIV transmission models in southern Africa noted that a substantial share of new transmissions is attributable to these 2 groups, whereas transmission from diagnosed individuals not yet on ART is generally more limited [7]. Empirical work also highlights a higher risk of disengagement among men and younger adults, with structural barriers contributing to retention gaps [8]. Together, these findings motivate analytic approaches that connect epidemic metrics to actionable gaps in diagnosis, retention, and re-engagement.

Population-based HIV Impact Assessment (PHIA) surveys, which are nationally representative and conducted in several African countries, provide a unique platform by integrating standardized sampling with biomarker-based measures of HIV status and viral load, as well as cascade indicators derived from self-report and laboratory evidence. PHIA diagnostic algorithms have demonstrated high performance across settings [9], and incidence estimation incorporates recent infection testing algorithms that integrate antiretroviral (ARV) drug detection to reduce false-recent classification [10].

In this study, we use sequential PHIA surveys from 6 high-HIV-burden countries (Eswatini, Lesotho, Malawi, Tanzania, Uganda, and Zimbabwe) to measure changes in HIV prevalence, cascade performance, population VLS (PVLS), PV, and incidence-based metrics, including IPR. We further characterize the distribution of residual viremia across cascade stages to identify priorities for targeted testing, retention, and re-engagement.

## METHODS

### Survey Design

Population-based HIV Impact Assessment surveys in Eswatini, Lesotho, Malawi, Tanzania, Uganda, and Zimbabwe used cross-sectional, 2-stage stratified cluster sampling to produce nationally representative estimates [11]. Enumeration areas were selected with probability proportional to size and stratified by region and urban-rural residence; within selected enumeration areas, households were selected by equal-probability systematic sampling. The number of households selected varied by survey rather than being fixed across countries and rounds. Institutional settings (eg, prisons and military barracks) were excluded. Fieldwork periods, response rates, and sample sizes are summarized in Supplementary Table 1.

### Participants and Analytic Age Range

Eligible individuals were household residents or overnight visitors who met country-specific age criteria, provided consent or assent, and completed interviews and biomarker collection. Children aged <15 years were excluded because pediatric data were not collected consistently across countries and rounds. All outcomes were restricted to adults aged 15–59 years to ensure comparability across countries and survey rounds. This age range was the only common adult analytic range across all 12 country-round surveys because the Lesotho Round 1 survey sampled adults aged 15–59 years.

### Procedures and Laboratory Measures

Standardized questionnaires captured sociodemographic characteristics, health history, and HIV service use. Venous blood was collected for HIV testing using national algorithms in the field; HIV-positive specimens were centrally confirmed, including Geenius supplemental testing, with Uganda applying centralized confirmation in Round 2 only. Confirmed HIV-positive specimens underwent centralized testing for recent infection classification (HIV-1 limiting antigen avidity enzyme immunoassay), HIV-1 viral load (Abbott RealTime or Roche COBAS), CD4+ cell count, and ARV drug detection in dried blood spots (efavirenz, nevirapine, lopinavir, and atazanavir; dolutegravir included in Round 2). Recent infection was defined using the PHIA recent infection testing algorithm (RITA), which integrates avidity results with viral load and ARV detection to reduce false-recent classifications [10].

### Outcomes

Human immunodeficiency virus prevalence was defined as the proportion of adults with confirmed HIV infection. Viral load suppression was defined as HIV-1 RNA <1000 copies/mL, and viremia as HIV-1 RNA ≥1000 copies/mL, among PWH with available viral load results. Population viral load suppression was defined as the proportion of PWH who were virally suppressed, regardless of awareness of HIV status or ART status, and is therefore distinct from the third 95, which is restricted to those aware of their HIV diagnosis and on ART. Population viremia was defined as the proportion of all adults in the population who were HIV-positive and viremic (viral load ≥1000 copies/mL). Population viremia was categorized into 3 mutually exclusive groups among viremic PWH: (1) undiagnosed, defined as unaware of HIV infection; (2) diagnosed but not on ART, including those not yet linked to ART and those with treatment interruption; and (3) diagnosed, on ART, but not suppressed.

Awareness was classified using standard PHIA definitions based on self-reported knowledge of prior diagnosis and/or evidence of ART, including ARV detection. Antiretroviral therapy use was defined as self-reported current ART and/or detectable ARVs. For categorization, missing awareness was classified as undiagnosed; among those classified as aware, missing ART status was classified as not on ART to ensure pathways summed to PV. Individuals with detectable ARVs were classified as “aware” and “on ART” regardless of self-report; consequently, the “undiagnosed and viremic” pathway includes only those with neither self-reported diagnosis nor evidence of ARV in their blood samples. Sensitivity analyses alternatively classified unknown ART status among those aware as on ART (Supplementary Table 5).

### Statistical Analysis

Analyses accounted for the complex survey design using sampling weights, clustering, and stratification (Stata 19). Weighted point estimates and 95% CIs for prevalence, cascade indicators, PVLS, and PV were calculated via Taylor series linearization. Biomarker-based outcomes were estimated among participants with the relevant laboratory result.

Human immunodeficiency virus incidence (aged 15–59 years) was estimated from microdata using the Kassanjee estimator applied to the RITA recency indicator [12]. We used PHIA-standard parameters (T = 1 year; false-recent rate = 0) under the full RITA that incorporates viral load and ARV detection [10]. Although this approach reduces false-recent classification, early treatment initiation may still affect recency assay performance in some settings [10]. The mean duration of recent infection (MDRI) was 130 days across Eswatini, Lesotho, Malawi, Tanzania, and Zimbabwe; in Uganda, round-specific MDRI values were used (153 days for Round 1; 156 days for Round 2), weighted for the HIV-1 subtype A/D distribution, consistent with survey specifications [13, 14]. Incidence is reported as a percentage per year; 95% CIs use survey-linearized variance for the recent proportion and the delta method for propagation (the lower bound is truncated at zero).

The IPR was calculated as (incidence/prevalence) × 100 within the same age group. Incidence-to-prevalence ratio 95% CIs were derived using the delta method on the log scale. As a descriptive benchmark, we compared IPR estimates with the commonly cited <3% threshold, proposed as a heuristic indicator of epidemic transition, and treated it as a reference point rather than an operational cutoff; cross-sectional incidence estimates may have limited precision for detecting modest short-term changes.

As a sensitivity analysis, we reclassified individuals who were aware of their HIV status but had unknown ART status as on ART and reassessed the PV category distribution (Supplementary Table 5).

To assess change between rounds, we estimated Round 2/Round 1 ratios for prevalence, cascade indicators, PVLS, and PV, and incidence rate ratios for incidence. CIs were constructed on the log scale using delta-method standard errors; 2-sided *P*-values came from Wald tests of the log ratio equal to zero. Missingness for key classification and biomarker variables is summarized as unweighted n (%) by country, round, and sex (Supplementary Table 4). Estimates based on <25 unweighted observations were suppressed, consistent with standard PHIA reporting conventions.

## RESULTS

Six countries (Eswatini, Lesotho, Malawi, Tanzania, Uganda, and Zimbabwe) completed sequential PHIA surveys in 2015–2017 (Round 1) and 2019–2023 (Round 2). The analytic sample comprised 113 970 adults aged 15–59 years in Round 1 (47 921 men [42.0%] and 66 049 women [58.0%]) and 112 445 in Round 2 (46 748 men [41.6%] and 65 697 women [58.4%]). Household response rates ranged from 82.8% to 96.7%, individual response rates from 80.4% to 97.9%, and blood draw response rates from 84.2% to 99.0% across surveys (Supplementary Table 1).

Human immunodeficiency virus prevalence among adults aged 15–59 years ranged from 5.0% (Tanzania) to 27.9% (Eswatini) in Round 1 and from 4.3% (Tanzania) to 25.5% (Eswatini) in Round 2 (Table 1). Women had a higher prevalence than men in all countries and both rounds (Supplementary Table 2). Round 2 HIV prevalence point estimates were lower than those in Round 1 in all 6 countries, although the estimates were similar in Uganda (6.2% vs 5.9%). (Table 1).

**Table 1.** HIV Prevalence, HIV Incidence, and Incidence-to-Prevalence Ratio by Country and Round (Aged 15–59 y)

Country	Round	HIV Prevalence, % (95% CI)	HIV Incidence, %/y (95% CI)	IPR, % (95% CI)
Eswatini	1	27.9 (26.4–29.4)	1.19 (.72–1.66)	4.3 (2.9–6.4)
	2	25.5 (24.2–26.7)	0.71 (.37–1.04)	2.8 (1.7–4.5)
Lesotho	1	25.6 (24.7–26.4)	1.11 (.69–1.53)	4.3 (3.0–6.3)
	2	23.5 (22.5–24.5)	0.50 (.26–.74)	2.1 (1.3–3.4)
Malawi	1	10.5 (9.9–11.1)	0.38 (.20–.55)	3.6 (2.3–5.7)
	2	8.8 (8.3–9.4)	0.23 (.11–.35)	2.6 (1.5–4.3)
Tanzania	1	5.0 (4.7–5.4)	0.26 (.16–.36)	5.3 (3.6–7.8)
	2	4.3 (4.0–4.6)	0.20 (.10–.29)	4.6 (2.8–7.3)
Uganda	1	6.2 (5.8–6.7)	0.42 (.27–.56)	6.7 (4.7–9.4)
	2	5.9 (5.4–6.4)	0.30 (.18–.42)	5.1 (3.4–7.6)
Zimbabwe	1	14.1 (13.4–14.7)	0.42 (.25–.59)	3.0 (2.0–4.4)
	2	13.1 (12.4–13.8)	0.42 (.22–.62)	3.2 (2.0–5.1)

Incidence was estimated from microdata using the Kassanjee estimator with PHIA-standard parameters (T = 1 y; FRR = 0 under the full RITA incorporating viral load and ARV detection). MDRI was 130 d for Eswatini, Lesotho, Malawi, Tanzania, and Zimbabwe; Uganda used 153 d (Round 1) and 156 d (Round 2). In Round 2, IPR CIs included the 3% epidemic-transition benchmark in 5 countries, while Uganda's interval remained above 3%. IPR values are rounded for display; the indicator is based on the unrounded point estimate.

Abbreviations: ARV, antiretroviral; FRR, false-recent rate; HIV, human immunodeficiency virus; IPR, incidence-to-prevalence ratio; MDRI, mean duration of recent infection; RITA, recent infection testing algorithm.

Incidence point estimates were numerically lower in Round 2 than in Round 1 in 5 countries, but 95% CIs were wide and largely overlapping across rounds, with the largest numerical decrease in Lesotho (Table 1). Among the 6 countries, IPRs ranged from 3.0% to 6.7% in Round 1 and from 2.1% to 5.1% in Round 2 (Table 1). In Round 2, Eswatini, Lesotho, and Malawi had IPR point estimates <3%, but 95% CIs included the 3% benchmark in all 3 countries (Table 1).

Testing and treatment cascade performance improved between rounds (Table 2). The proportion of PWH who were aware of their status was higher in Round 2 than in Round 1 in all 6 countries, but no country reached 95% awareness in Round 2; Eswatini had the highest level (93.5%) (Table 2). Antiretroviral therapy coverage among those aware of HIV status exceeded 95% in all 6 countries in Round 2 (95.9%–97.7%). Viral load suppression among those on ART increased in all countries, and the third 95 UNAIDS target for VLS was met in Eswatini (95.9%) and Malawi (96.9%) in Round 2. Population viral load suppression increased in all 6 countries, rising from 52.0%–72.4% in Round 1 to 74.5%–88.0% in Round 2 (Table 2). Population viremia declined in all 6 countries; across countries, it ranged from 2.4% to 8.3% in Round 1 and from 1.0% to 4.7% in Round 2 (Table 3; Figure 2*A*).

**Table 2.** HIV Testing and Treatment Cascade and Population Viral Load Suppression by Country and Round (Aged 15–59 y)

Country	Round	Aware, % (95% CI)	On ART Among Those Aware, % (95% CI)	VLS Among Those on ART, % (95% CI)	Population VLS, % (95% CI)
Eswatini	1	86.8 (85.6–88.1)	88.3 (86.8–89.8)	91.0 (90.0–92.1)	72.4 (70.5–74.2)
	2	93.5 (92.4–94.6)	97.2 (96.4–97.9)	95.9 (95.0–96.7)	88.0 (86.7–89.4)
Lesotho	1	81.0 (79.7–82.3)	91.8 (90.6–93.1)	87.7 (86.2–89.2)	67.6 (65.8–69.4)
	2	89.6 (88.4–90.9)	96.6 (95.9–97.4)	90.8 (89.6–92.0)	79.9 (78.2–81.6)
Malawi	1	76.6 (74.5–78.7)	91.3 (89.7–92.8)	91.2 (89.2–93.1)	68.0 (65.8–70.2)
	2	88.4 (86.7–90.0)	97.7 (97.0–98.4)	96.9 (96.0–97.8)	86.9 (85.3–88.5)
Tanzania	1	62.2 (58.8–65.5)	93.5 (91.7–95.2)	87.0 (84.3–89.6)	52.0 (48.8–55.2)
	2	81.9 (79.2–84.6)	97.5 (96.5–98.6)	94.0 (92.3–95.6)	76.9 (74.3–79.6)
Uganda	1	72.1 (69.6–74.6)	90.3 (88.3–92.2)	83.9 (81.7–86.1)	59.3 (56.5–62.1)
	2	80.5 (77.9–83.0)	95.9 (94.6–97.2)	91.9 (90.2–93.6)	74.5 (71.8–77.2)
Zimbabwe	1	76.6 (74.8–78.5)	88.2 (86.9–89.5)	84.9 (83.1–86.7)	59.1 (56.9–61.2)
	2	86.4 (84.7–88.1)	96.7 (95.8–97.5)	89.7 (88.3–91.1)	76.1 (74.1–78.1)

Population VLS is the proportion of all people with HIV who were virally suppressed.

Abbreviations: ART, antiretroviral therapy; HIV, human immunodeficiency virus; VLS, viral load suppression (HIV-1 RNA <1000 copies/mL).

**Table 3.** Population Viremia Categorization by Country and Round (Aged 15–59 y)

Country	Round	PV, % (95% CI)	Undiagnosed and Viremic, % (95% CI)	Diagnosed, No ART and Viremic, % (95% CI)	On ART, Not Suppressed, % (95% CI)	Share Undiag., %	Share Diag. No ART, %	Share on ART, %
Eswatini	1	7.7 (7.0–8.5)	3.29 (2.87–3.70)	2.52 (2.13–2.91)	1.91 (1.64–2.18)	42.6	32.6	24.8
	2	3.0 (2.7–3.4)	1.45 (1.17–1.73)	0.64 (.46–.82)	0.96 (.75–1.16)	47.6	21.0	31.4
Lesotho	1	8.3 (7.7–8.8)	4.38 (4.04–4.73)	1.55 (1.29–1.81)	2.34 (2.03–2.65)	53.0	18.7	28.3
	2	4.7 (4.3–5.2)	2.19 (1.90–2.49)	0.66 (.51–.81)	1.87 (1.62–2.13)	46.4	13.9	39.7
Malawi	1	3.4 (3.1–3.6)	2.04 (1.81–2.27)	0.67 (.54–.79)	0.65 (.50–.79)	60.9	19.8	19.2
	2	1.2 (1.0–1.3)	0.77 (.65–.89)	0.15 (.10–.20)	0.24 (.17–.30)	66.4	13.2	20.3
Tanzania	1	2.4 (2.2–2.6)	1.74 (1.54–1.94)	0.31 (.23–.39)	0.36 (.28–.44)	72.2	12.7	15.1
	2	1.0 (.9–1.1)	0.71 (.60–.82)	0.08 (.04–.11)	0.21 (.15–.27)	71.4	7.6	21.0
Uganda	1	2.5 (2.3–2.8)	1.48 (1.30–1.66)	0.41 (.32–.50)	0.66 (.56–.75)	58.1	16.2	25.8
	2	1.5 (1.3–1.7)	0.96 (.82–1.11)	0.17 (.11–.23)	0.37 (.29–.45)	64.1	11.3	24.5
Zimbabwe	1	5.8 (5.3–6.2)	3.12 (2.82–3.43)	1.20 (1.05–1.35)	1.43 (1.25–1.62)	54.3	20.8	24.9
	2	3.1 (2.8–3.5)	1.67 (1.43–1.91)	0.34 (.25–.43)	1.13 (0.97–1.29)	53.1	10.8	36.1

For the viremia decomposition, viremic people with HIV with missing awareness status are classified as undiagnosed to ensure mutually exclusive categories that sum to total PV. Share columns give each pathway's percentage contribution to total PV.

Abbreviations: ART, antiretroviral therapy; HIV, human immunodeficiency virus; PV, population viremia.

**Figure 1.**
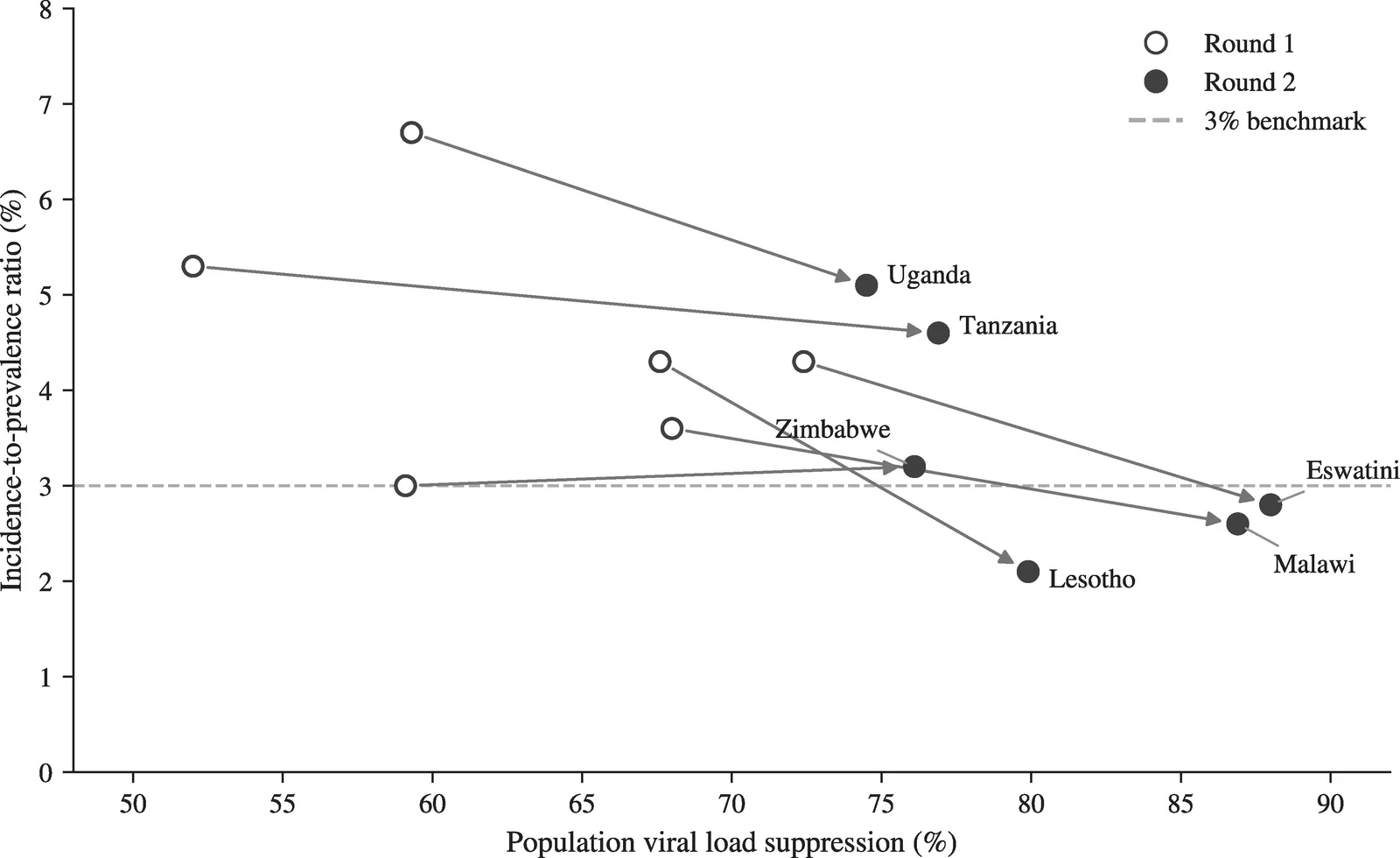
Population viral load suppression (PVLS) and the incidence-to-prevalence ratio, by country and survey round. For each country, the Round 1 estimate (open circle) is joined by an arrow to the Round 2 estimate (filled circle). The *x*-axis shows PVLS (%), and the *y*-axis shows the incidence-to-prevalence ratio (IPR, %). The dashed horizontal line marks the 3% IPR benchmark. Both indicators cover adults aged 15–59 y.

Figure 1 plots each country's Round 1 to Round 2 trajectory for PVLS and the IPR. Population viral load suppression increased in all 6 countries, whereas IPR point estimates declined in 5 countries and were similar in Zimbabwe; in Round 2, Uganda and Tanzania remained above 4% (Figure 1; Table 1).

**Figure 2.**
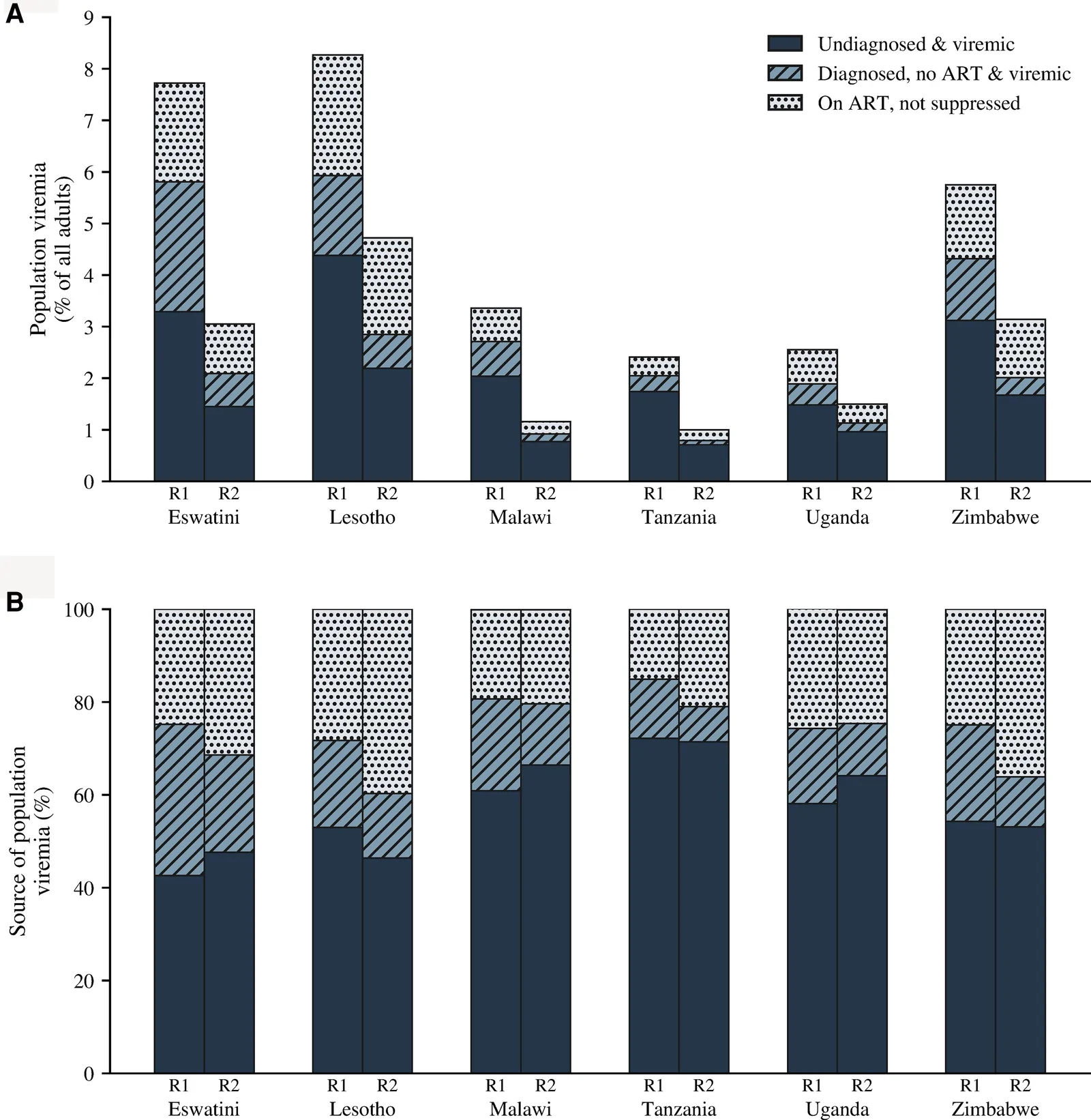
Population viremia and its composition, by country and survey round. *A*, Absolute population viremia (percentage of all adults aged 15–59 y), partitioned into 3 mutually exclusive pathways among viremic people with human immunodeficiency virus (HIV)—undiagnosed and viremic; diagnosed but not on antiretroviral therapy (ART) and viremic; on ART but not suppressed—summing to total population viremia. *B*, Proportional composition (%) across the same 3 pathways. R1, Round 1; R2, Round 2. Categories are distinguished by color and fill pattern for grayscale legibility.

Population viremia categories are shown in Table 3 and Figure 2*A* and 2*B*. Declines in PV were largest in Eswatini (7.7% to 3.0%), Lesotho (8.3% to 4.7%), and Zimbabwe (5.8% to 3.1%) (Table 3; Figure 2*A*). In Round 2, undiagnosed HIV infection accounted for most residual PV in Tanzania (71.4%), Malawi (66.4%), Uganda (64.1%), and Zimbabwe (53.1%) and remained substantial in Eswatini (47.6%) and Lesotho (46.4%). In Round 2, the share of residual PV attributed to those on ART but not suppressed exceeded the share attributed to those diagnosed but not on ART in all 6 countries (Table 3; Figure 2*B*). The share of PV attributed to people on ART but not suppressed increased between rounds in 5 countries, with the largest increases in Lesotho and Zimbabwe (Table 3; Figure 2*B*). Missingness for key classification and biomarker variables was low across countries and rounds (Supplementary Table 4). In sensitivity analyses, results were similar to the primary classification (Supplementary Table 5).

## DISCUSSION

In this comparative analysis of sequential, nationally representative PHIA surveys from 6 high-HIV-burden countries, we observed improvements throughout the HIV diagnosis and treatment cascade, accompanied by declines in PV. These patterns are consistent with the programmatic intent of the 95–95–95 framework to reduce morbidity and transmission by expanding diagnosis, sustaining ART, and achieving viral suppression [2, 15]. However, changes in incidence-based measures were heterogeneous across settings, underscoring that improvements in cascade indicators alone are insufficient to infer corresponding reductions in HIV acquisition [4, 5]. This pattern is consistent with 2-round PHIA evidence among adolescents, in whom gains in knowledge of HIV status and ART coverage were not accompanied by clear declines in incidence [16].

Population viremia may be a useful marker of epidemic momentum because it captures the population-level burden of unsuppressed infection, which is directly relevant to transmission potential; declines in PV therefore complement improvements in PVLS by reflecting reductions in viremia across the entire population rather than just suppression among PWH [6]. Categorizing PV into mutually exclusive pathways adds operational specificity by identifying where residual viremia is concentrated along the cascade, analogous to prevention of mother-to-child transmission (PMTCT) cascade analyses that identify actionable gaps along the continuum of care [17]. This framing is consistent with HIV transmission modeling in southern Africa, which attributes a substantial share of onward transmission to undiagnosed individuals and people with interrupted ART, and comparatively little to individuals diagnosed but not yet on ART [7]. Compositional shifts should be interpreted alongside the absolute decline in total PV: the share attributable to people on ART but not suppressed increased in some settings even as total PV declined and therefore should not be interpreted as evidence of worsening treatment outcomes without pathway-specific analysis. Because pathway assignment relied on self-report supplemented by ARV detection, the “diagnosed but not on ART” pathway may include both people who had never initiated ART and people who had initiated and subsequently stopped ART but whose prior ART use was not captured in the interview. Likewise, people who did not disclose a prior HIV diagnosis and had no detectable ARVs may have been classified as undiagnosed. These histories could not be distinguished directly. In Round 2, the share of residual PV attributable to people on ART but not suppressed exceeded the share attributable to those diagnosed but not on ART in all 6 countries, indicating that programs should combine rapid linkage and low-barrier re-engagement with adherence support and regimen optimization.

Round 2 fieldwork was staggered across countries (Supplementary Table 1) and overlapped with the broader transition to dolutegravir-based first-line regimens in many settings. Consequently, countries were surveyed at different stages of implementation. Dolutegravir-based regimens, which offer rapid viral suppression, favorable tolerability, and a high genetic barrier to resistance [18], may have contributed to higher viral suppression in Round 2; however, because individual-level regimen exposure and the timing and extent of rollout were not measured consistently across surveys, we could not quantify the contribution of dolutegravir or distinguish it from other concurrent program changes.

More broadly, between survey rounds, countries adopted and scaled several other HIV program recommendations at different times, including expanded routine and targeted HIV testing, universal test-and-treat, differentiated service delivery, and viral load monitoring [19]. Accordingly, the observed changes cannot be attributed to any single intervention and should be interpreted in the context of multiple concurrent program changes. Expansion of treatment eligibility and ART enrollment may also have altered the size and composition of the population receiving ART. Because PHIA surveys are cross-sectional and do not follow program cohorts, we could not determine whether such changes affected treatment interruption or viral nonsuppression.

Our findings highlight the importance of measurement quality when interpreting incidence-based metrics. Because cross-sectional incidence estimates derived from recency testing depend on assay-based classification of recent infection and on the assumptions of the incidence estimator, between-round and cross-country comparisons of incidence and the IPR should be interpreted cautiously, particularly when CIs are wide and overlap [10, 12].

These findings support strengthening routine viral load monitoring and ensuring timely clinical action following an unsuppressed result [19]. Population survey estimates should be triangulated with routine measures of viral load testing coverage, result turnaround times, viral suppression, and clinical response to distinguish gaps in testing access, treatment continuity, and regimen response.

From a program implementation perspective, consistently lower PVLS among men (Supplementary Table 3) supports person-centered service delivery with particular attention to men. A mixed-methods cohort in South Africa documented substantial disengagement during the first 6 months of ART, with greater risk among men and younger clients [8]. Differentiated service delivery models generally achieve retention and viral suppression comparable to standard care among clinically stable clients [20] and may be particularly important where geographic access to facility-based care is limited [21]. Client satisfaction and preferences should also inform service design [22], while structured re-engagement approaches such as “Welcome Services” can reduce administrative barriers and expedite ART restart after treatment interruption [23]. Together, this evidence reinforces the need to match service models to individual circumstances.

Incidence-to-prevalence ratio thresholds should be treated as reference points rather than operational cutoffs. The commonly cited IPR <3% benchmark has been proposed as a heuristic indicator of epidemic transition, reflecting low incidence relative to HIV prevalence; however, its interpretation depends on survival and demographic changes and should be considered alongside other indicators [4, 5]. In our analysis, wide and largely overlapping CIs limited our ability to detect modest but potentially meaningful between-round changes in incidence and IPR (Table 1). Consistent with broader guidance on measuring epidemic trends and progress [24, 25], we therefore interpreted incidence and IPR alongside cascade indicators and PV rather than assigning epidemic-transition status based on a single metric. Where residual viremia is concentrated primarily among undiagnosed individuals, closing the diagnostic gap may require intensified use of evidence-based testing strategies, including index and partner testing, targeted community- and venue-based testing, and secondary distribution of HIV self-tests [19, 26].

This study has several strengths. PHIA surveys provide rigorous, comparable, nationally representative data with biomarker confirmation and standardized protocols that support cross-country assessment of HIV prevalence, cascade outcomes, PV, and incidence [11]. Standardized HIV testing algorithms and laboratory confirmation procedures further strengthen confidence in biomarker-based measures and cross-survey comparability [9].

Limitations include the cross-sectional design, which precludes causal attribution of changes between rounds to specific interventions, and the potential for residual bias from self-reported measures and differential nonresponse despite weighting adjustments. Incidence estimates derived from cross-sectional recency testing, and therefore IPR estimates, depend on assumptions about recent-infection classification and assay performance; wide and largely overlapping CIs limited the precision of between-round comparisons and the ability to detect modest short-term changes. Cascade measures also warrant cautious interpretation because detectable ARVs were used to classify awareness and ART use, and dolutegravir was added to the ARV detection panel in Round 2; this difference may have affected cross-round classification. Population viremia pathway classification may also have been affected by incomplete disclosure of prior diagnosis or ART use among participants with no detectable ARVs. Because the PHIA rounds were repeated cross-sectional surveys rather than longitudinal cohorts, between-round differences may also reflect changes in population composition, including survival, migration, and aging of PWH beyond age 59 years; restricting both rounds to 15–59 years of age improved comparability but limits inference about the total burden among older adults. Finally, Round 2 fieldwork occurred at different phases of the COVID-19 pandemic, including pandemic-related pauses in Malawi and Uganda (Supplementary Table 1); these timing differences, together with potential misclassification within the viremia decomposition framework, may have contributed to heterogeneity in observed cross-round patterns.

In summary, sequential PHIA surveys across 6 countries indicate improvements in diagnosis, treatment, and viral suppression, together with declines in PV, but provide uneven and uncertain evidence for reductions in HIV acquisition. Our findings support a combined approach that uses cascade indicators, PV, and incidence-based measures to identify whether residual viremia is concentrated among undiagnosed people, diagnosed people not receiving ART, or people receiving ART without viral suppression. Continued progress will likely require maintaining high treatment coverage, intensifying targeted testing strategies, strengthening routine viral load monitoring and timely clinical action after unsuppressed results, and implementing person-centered service-delivery models, including structured re-engagement services, to support rapid return to and sustained engagement in care among people who experience treatment interruption [15, 19, 23, 27].

## Supplementary Material

ofag482_Supplementary_Data

## Supplementary Data

Supplementary materials are available at *Open Forum Infectious Diseases* online. Consisting of data provided by the authors to benefit the reader, the posted materials are not copyedited and are the sole responsibility of the authors, so questions or comments should be addressed to the corresponding author.
