# Supplementary material for: Population HIV Viremia and Epidemic Transition: Findings From Sequential Surveys in 6 Sub-Saharan African Countries
Source: Open Forum Infect Dis. 2026 Aug 8;13(9):ofag482. doi: 10.1093/ofid/ofag482 (PMC13542725; doi:10.1093/ofid/ofag482)
Supplement: Supplementary file 1 [file ofag482_supplementary_data.docx]

**Table S1. Survey characteristics, fieldwork periods, response rates, and analytic sample sizes (ages 15–59 years).**

| **Country** | **Fieldwork period** | **Household response, %** | **Individual response, female, %** | **Individual response, male, %** | **Blood draw response, female, %** | **Blood draw response, male, %** | **Analytic sample, n** |
| --- | --- | --- | --- | --- | --- | --- | --- |
| Eswatini | Aug 2016–Mar 2017 | 84.5 | 93.7 | 86.7 | 94.7 | 91.6 | 9,559 |
| Eswatini | May–Nov 2021 | 82.8 | 90.8 | 80.4 | 92.8 | 91.2 | 9,768 |
| Lesotho | Nov 2016–May 2017 | 93.0 | 95.1 | 86.8 | 91.3 | 88.2 | 11,682 |
| Lesotho | Dec 2019–Mar 2020 | 93.1 | 96.1 | 89.8 | 93.3 | 92.1 | 12,718 |
| Malawi | Nov 2015–Aug 2016 | 90.2 | 92.9 | 81.5 | 87.8 | 87.0 | 16,700 |
| Malawi | Jan 2020–Apr 2021* | 91.7 | 91.7 | 83.5 | 84.2 | 85.8 | 20,503 |
| Tanzania | Oct 2016–Aug 2017† | 94.8 | 93.2 | 88.5 | 95.2 | 94.4 | 28,352 |
| Tanzania | Nov 2022–Mar 2023 | 92.7 | 93.0 | 87.3 | 93.0 | 92.6 | 29,410 |
| Uganda | Aug 2016–Mar 2017 | 96.7 | 97.9 | 94.0 | 99.0 | 98.5 | 28,041 |
| Uganda | Feb 2020–Mar 2021‡ | 95.9 | 96.4 | 91.5 | 97.9 | 97.5 | 23,148 |
| Zimbabwe | Oct 2015–Aug 2016 | 83.9 | 93.9 | 82.3 | 91.8 | 90.2 | 19,636 |
| Zimbabwe | Nov 2019–Mar 2020 | 88.6 | 94.5 | 86.5 | 94.2 | 92.8 | 16,898 |

Abbreviations: PHIA, Population-based HIV Impact Assessment; SHIMS, Eswatini HIV Incidence Measurement Survey; THIS, Tanzania HIV Impact Survey.

Notes: Response rates and fieldwork periods were abstracted from PHIA final reports. Analytic sample size is the unweighted number of adults aged 15–59 years with non-missing HIV status and valid survey design variables. * Malawi Round 2 fieldwork paused from April 2020 through March 2021 because of the COVID-19 pandemic. † The Tanzania THIS 2016–2017 final report identifies October 2016 as the beginning of fieldwork in its overview sections and November 2016 as the beginning of data collection; the table follows the reported fieldwork period. ‡ Uganda Round 2 fieldwork paused from March through October 2020 because of the COVID-19 pandemic. For Eswatini, Round 1 was SHIMS2 and Round 2 was SHIMS3; the survey numbering reflects an earlier national SHIMS conducted in 2011.

**Table S2. HIV prevalence, HIV incidence, and HIV incidence-to-prevalence ratio by country, sex, and round (ages 15–59 years).**

| **Country** | **Round** | **Sex** | **HIV prevalence, % (95% CI)** | **HIV incidence, % per year (95% CI)** | **Incidence-to-prevalence ratio, % (95% CI)** |
| --- | --- | --- | --- | --- | --- |
| Eswatini | 1 | Male | 20.4 (18.6–22.1) | 0.80 (0.17–1.44) | 3.9 (1.8–8.7) |
| Eswatini | 1 | Female | 34.2 (32.6–35.9) | 1.58 (0.89–2.28) | 4.6 (3.0–7.2) |
| Eswatini | 2 | Male | 18.2 (16.7–19.7) | 0.18 (0.00–0.44) | 1.0 (0.2–4.2) |
| Eswatini | 2 | Female | 32.4 (30.6–34.2) | 1.31 (0.66–1.95) | 4.0 (2.5–6.6) |
| Lesotho | 1 | Male | 20.8 (19.6–22.0) | 1.01 (0.42–1.60) | 4.9 (2.7–8.8) |
| Lesotho | 1 | Female | 30.4 (29.2–31.5) | 1.22 (0.72–1.73) | 4.0 (2.7–6.1) |
| Lesotho | 2 | Male | 17.8 (16.7–19.0) | 0.31 (0.04–0.58) | 1.7 (0.7–4.2) |
| Lesotho | 2 | Female | 29.3 (28.0–30.5) | 0.72 (0.34–1.11) | 2.5 (1.4–4.2) |
| Malawi | 1 | Male | 8.4 (7.8–9.1) | 0.24 (0.07–0.40) | 2.8 (1.4–5.7) |
| Malawi | 1 | Female | 12.5 (11.6–13.3) | 0.51 (0.21–0.82) | 4.1 (2.3–7.5) |
| Malawi | 2 | Male | 6.9 (6.3–7.6) | 0.13 (0.00–0.30) | 1.9 (0.6–6.5) |
| Malawi | 2 | Female | 10.6 (10.0–11.2) | 0.32 (0.15–0.49) | 3.0 (1.7–5.1) |
| Tanzania | 1 | Male | 3.5 (3.1–3.8) | 0.17 (0.05–0.30) | 5.0 (2.4–10.4) |
| Tanzania | 1 | Female | 6.5 (6.0–7.0) | 0.35 (0.19–0.51) | 5.4 (3.4–8.5) |
| Tanzania | 2 | Male | 2.9 (2.6–3.2) | 0.12 (0.01–0.23) | 4.2 (1.7–10.1) |
| Tanzania | 2 | Female | 5.6 (5.2–6.1) | 0.27 (0.12–0.41) | 4.8 (2.7–8.3) |
| Uganda | 1 | Male | 4.7 (4.3–5.1) | 0.36 (0.16–0.56) | 7.7 (4.4–13.5) |
| Uganda | 1 | Female | 7.7 (7.1–8.2) | 0.47 (0.29–0.65) | 6.1 (4.1–9.0) |
| Uganda | 2 | Male | 4.2 (3.7–4.7) | 0.20 (0.05–0.36) | 4.9 (2.2–10.6) |
| Uganda | 2 | Female | 7.4 (6.8–8.1) | 0.39 (0.22–0.57) | 5.3 (3.3–8.3) |
| Zimbabwe | 1 | Male | 11.8 (11.0–12.7) | 0.31 (0.08–0.54) | 2.6 (1.3–5.5) |
| Zimbabwe | 1 | Female | 16.1 (15.4–16.9) | 0.52 (0.27–0.77) | 3.2 (2.0–5.2) |
| Zimbabwe | 2 | Male | 10.2 (9.4–11.0) | 0.21 (0.02–0.41) | 2.1 (0.8–5.3) |
| Zimbabwe | 2 | Female | 15.9 (14.9–16.8) | 0.62 (0.30–0.94) | 3.9 (2.3–6.5) |

**Table S3. HIV testing and treatment cascade and population viral load suppression by country, sex, and round (ages 15–59 years).**

| **Country** | **Round** | **Sex** | **Aware of HIV status, % (95% CI)** | **On ART among those aware, % (95% CI)** | **VLS among those on ART, % (95% CI)** | **Population VLS, % (95% CI)** |
| --- | --- | --- | --- | --- | --- | --- |
| Eswatini | 1 | Male | 79.2 (76.4–81.9) | 89.3 (86.7–91.9) | 89.7 (87.2–92.2) | 65.7 (62.6–68.7) |
| Eswatini | 1 | Female | 90.6 (89.2–92.0) | 87.9 (86.1–89.6) | 91.6 (90.3–93.0) | 75.7 (73.5–77.9) |
| Eswatini | 2 | Male | 91.0 (88.9–93.0) | 95.6 (94.0–97.2) | 96.2 (94.9–97.6) | 84.9 (82.4–87.5) |
| Eswatini | 2 | Female | 94.8 (93.7–96.0) | 97.9 (97.2–98.7) | 95.7 (94.7–96.6) | 89.6 (88.3–91.0) |
| Lesotho | 1 | Male | 76.6 (74.3–78.9) | 91.6 (89.7–93.5) | 87.7 (85.1–90.2) | 63.4 (60.5–66.3) |
| Lesotho | 1 | Female | 84.0 (82.4–85.7) | 92.0 (90.5–93.5) | 87.7 (86.0–89.4) | 70.5 (68.4–72.6) |
| Lesotho | 2 | Male | 87.3 (84.9–89.7) | 95.6 (94.3–96.9) | 89.2 (86.9–91.4) | 75.5 (72.7–78.4) |
| Lesotho | 2 | Female | 91.1 (89.8–92.3) | 97.3 (96.5–98.0) | 91.7 (90.3–93.1) | 82.5 (80.8–84.3) |
| Malawi | 1 | Male | 71.2 (67.4–75.0) | 88.6 (85.5–91.8) | 90.0 (86.6–93.5) | 60.7 (56.8–64.6) |
| Malawi | 1 | Female | 80.1 (77.8–82.4) | 92.8 (91.1–94.5) | 91.8 (89.7–93.9) | 72.7 (70.0–75.3) |
| Malawi | 2 | Male | 85.3 (82.4–88.1) | 97.1 (95.6–98.6) | 97.0 (95.5–98.4) | 85.2 (82.4–87.9) |
| Malawi | 2 | Female | 90.2 (88.5–92.0) | 98.1 (97.3–98.8) | 96.9 (95.8–98.0) | 88.0 (86.2–89.8) |
| Tanzania | 1 | Male | 53.2 (47.5–58.9) | 89.6 (85.5–93.6) | 83.6 (78.1–89.2) | 41.2 (36.2–46.3) |
| Tanzania | 1 | Female | 66.8 (63.3–70.2) | 95.1 (93.4–96.8) | 88.3 (85.3–91.2) | 57.4 (54.0–60.9) |
| Tanzania | 2 | Male | 76.2 (70.9–81.4) | 96.0 (93.5–98.6) | 92.8 (89.7–95.9) | 70.0 (64.7–75.2) |
| Tanzania | 2 | Female | 84.6 (81.9–87.2) | 98.2 (97.3–99.1) | 94.4 (92.8–96.1) | 80.3 (77.5–83.0) |
| Uganda | 1 | Male | 66.7 (62.5–70.9) | 86.9 (83.1–90.7) | 82.1 (77.3–86.9) | 53.5 (48.8–58.2) |
| Uganda | 1 | Female | 75.1 (72.2–78.0) | 91.9 (89.8–94.1) | 84.7 (82.1–87.3) | 62.5 (59.4–65.6) |
| Uganda | 2 | Male | 75.0 (70.2–79.8) | 94.4 (91.8–97.0) | 90.6 (87.4–93.8) | 68.2 (63.5–72.9) |
| Uganda | 2 | Female | 83.3 (80.8–85.8) | 96.6 (95.3–97.9) | 92.4 (90.5–94.4) | 77.8 (74.9–80.6) |
| Zimbabwe | 1 | Male | 71.4 (68.2–74.6) | 87.4 (84.9–89.9) | 81.9 (78.6–85.2) | 52.5 (49.2–55.7) |
| Zimbabwe | 1 | Female | 80.1 (78.2–82.0) | 88.6 (87.1–90.2) | 86.7 (84.9–88.5) | 63.4 (61.2–65.7) |
| Zimbabwe | 2 | Male | 83.3 (80.1–86.5) | 95.4 (93.7–97.2) | 88.3 (85.5–91.0) | 71.3 (67.6–75.0) |
| Zimbabwe | 2 | Female | 88.2 (86.6–89.8) | 97.4 (96.5–98.2) | 90.5 (88.9–92.0) | 79.0 (76.9–81.0) |

**Table S4. Sample sizes and missing data for key classification and biomarker variables among adults aged 15–59 years, by country, sex, and survey round.**

| **Country** | **Sex** | **n adults (Round 1 / Round 2)** | **n people with HIV (Round 1 / Round 2)** | **Viral load missing (Round 1 / Round 2)** | **CD4 missing (Round 1 / Round 2)** | **Awareness missing (Round 1 / Round 2)** | **Recency missing (Round 1 / Round 2)** | **Antiretroviral therapy status missing (Round 1 / Round 2)** |
| --- | --- | --- | --- | --- | --- | --- | --- | --- |
| Eswatini | Total | 9,559 / 9,768 | 2,791 / 2,616 | 0 (0.0%) / 11 (0.4%) | 3 (0.1%) / 5 (0.2%) | 1 (0.0%) / 0 (0.0%) | 1 (0.0%) / 13 (0.5%) | 5 (0.2%) / 9 (0.4%) |
|  | Male | 4,023 / 4,133 | 871 / 750 | 0 (0.0%) / 4 (0.5%) | 0 (0.0%) / 0 (0.0%) | 0 (0.0%) / 0 (0.0%) | 0 (0.0%) / 6 (0.8%) | 2 (0.3%) / 3 (0.4%) |
|  | Female | 5,536 / 5,635 | 1,920 / 1,866 | 0 (0.0%) / 7 (0.4%) | 3 (0.2%) / 5 (0.3%) | 1 (0.1%) / 0 (0.0%) | 1 (0.1%) / 7 (0.4%) | 3 (0.2%) / 6 (0.3%) |
| Lesotho | Total | 11,682 / 12,718 | 3,199 / 3,239 | 2 (0.1%) / 4 (0.1%) | 8 (0.3%) / 2 (0.1%) | 2 (0.1%) / 0 (0.0%) | 2 (0.1%) / 4 (0.1%) | 2 (0.1%) / 3 (0.1%) |
|  | Male | 4,762 / 5,313 | 1,019 / 1,032 | 0 (0.0%) / 1 (0.1%) | 3 (0.3%) / 0 (0.0%) | 0 (0.0%) / 0 (0.0%) | 0 (0.0%) / 1 (0.1%) | 1 (0.1%) / 0 (0.0%) |
|  | Female | 6,920 / 7,405 | 2,180 / 2,207 | 2 (0.1%) / 3 (0.1%) | 5 (0.2%) / 2 (0.1%) | 2 (0.1%) / 0 (0.0%) | 2 (0.1%) / 3 (0.1%) | 1 (0.1%) / 3 (0.1%) |
| Malawi | Total | 16,700 / 20,503 | 2,157 / 2,239 | 7 (0.3%) / 3 (0.1%) | 25 (1.2%) / 1 (0.0%) | 3 (0.1%) / 0 (0.0%) | 4 (0.2%) / 3 (0.1%) | 7 (0.4%) / 3 (0.1%) |
|  | Male | 6,956 / 8,656 | 680 / 678 | 3 (0.4%) / 0 (0.0%) | 11 (1.6%) / 0 (0.0%) | 1 (0.1%) / 0 (0.0%) | 3 (0.4%) / 0 (0.0%) | 2 (0.4%) / 0 (0.0%) |
|  | Female | 9,744 / 11,847 | 1,477 / 1,561 | 4 (0.3%) / 3 (0.2%) | 14 (0.9%) / 1 (0.1%) | 2 (0.1%) / 0 (0.0%) | 1 (0.1%) / 3 (0.2%) | 5 (0.4%) / 3 (0.2%) |
| Tanzania | Total | 28,352 / 29,410 | 1,709 / 1,625 | 1 (0.1%) / 1 (0.1%) | 7 (0.4%) / 4 (0.2%) | 0 (0.0%) / 0 (0.0%) | 0 (0.0%) / 1 (0.1%) | 48 (4.5%) / 0 (0.0%) |
|  | Male | 12,271 / 12,408 | 517 / 461 | 1 (0.2%) / 1 (0.2%) | 4 (0.8%) / 3 (0.7%) | 0 (0.0%) / 0 (0.0%) | 0 (0.0%) / 1 (0.2%) | 12 (4.3%) / 0 (0.0%) |
|  | Female | 16,081 / 17,002 | 1,192 / 1,164 | 0 (0.0%) / 0 (0.0%) | 3 (0.3%) / 1 (0.1%) | 0 (0.0%) / 0 (0.0%) | 0 (0.0%) / 0 (0.0%) | 36 (4.5%) / 0 (0.0%) |
| Uganda | Total | 28,041 / 23,148 | 1,709 / 1,388 | 0 (0.0%) / 0 (0.0%) | 23 (1.3%) / 4 (0.3%) | 1 (0.1%) / 2 (0.1%) | 0 (0.0%) / 1 (0.1%) | 2 (0.2%) / 1 (0.1%) |
|  | Male | 11,903 / 9,625 | 542 / 422 | 0 (0.0%) / 0 (0.0%) | 10 (1.8%) / 3 (0.7%) | 0 (0.0%) / 0 (0.0%) | 0 (0.0%) / 0 (0.0%) | 0 (0.0%) / 0 (0.0%) |
|  | Female | 16,138 / 13,523 | 1,167 / 966 | 0 (0.0%) / 0 (0.0%) | 13 (1.1%) / 1 (0.1%) | 1 (0.1%) / 2 (0.2%) | 0 (0.0%) / 1 (0.1%) | 2 (0.2%) / 1 (0.1%) |
| Zimbabwe | Total | 19,636 / 16,898 | 3,238 / 2,642 | 5 (0.2%) / 1 (0.0%) | 3 (0.1%) / 4 (0.2%) | 1 (0.0%) / 0 (0.0%) | 5 (0.2%) / 5 (0.2%) | 1 (0.0%) / 0 (0.0%) |
|  | Male | 8,006 / 6,613 | 1,085 / 781 | 2 (0.2%) / 0 (0.0%) | 0 (0.0%) / 0 (0.0%) | 1 (0.1%) / 0 (0.0%) | 2 (0.2%) / 2 (0.3%) | 1 (0.1%) / 0 (0.0%) |
|  | Female | 11,630 / 10,285 | 2,153 / 1,861 | 3 (0.1%) / 1 (0.1%) | 3 (0.1%) / 4 (0.2%) | 0 (0.0%) / 0 (0.0%) | 3 (0.1%) / 3 (0.2%) | 0 (0.0%) / 0 (0.0%) |

Abbreviations: CD4, cluster of differentiation 4; PHIA, Population-based HIV Impact Assessment.

Notes: Missing data are shown as n (%). Percentages for viral load, CD4, awareness, and recency missingness are calculated among people with HIV; percentages for antiretroviral therapy status missingness are calculated among people classified as aware of their HIV status. HIV status had 0% missingness across all surveys. Shaded rows indicate totals for each country. Antiretroviral therapy status missingness was highest in Tanzania Round 1 (4.5%), primarily due to questionnaire skip patterns.

**Table S5. Sensitivity analysis for population viremia decomposition: alternative classification of unknown antiretroviral therapy status among those aware as on antiretroviral therapy.**

| **Country** | **Round** | **Undiagnosed and viremic, % of population viremia (primary)** | **Diagnosed but not on antiretroviral therapy and viremic, % of population viremia (primary)** | **On antiretroviral therapy but not suppressed, % of population viremia (primary)** | **Change in undiagnosed share, pp (sensitivity minus primary)** | **Change in diagnosed not on antiretroviral therapy share, pp (sensitivity minus primary)** | **Change in on antiretroviral therapy not suppressed share, pp (sensitivity minus primary)** |
| --- | --- | --- | --- | --- | --- | --- | --- |
| Eswatini | 1 | 42.6 | 32.6 | 24.8 | 0 | -0.6 | 0.6 |
|  | 2 | 47.6 | 21.0 | 31.4 | 0 | 0 | 0 |
| Lesotho | 1 | 53.0 | 18.7 | 28.3 | 0 | -0.2 | 0.2 |
|  | 2 | 46.4 | 13.9 | 39.7 | 0 | 0 | 0 |
| Malawi | 1 | 60.9 | 19.8 | 19.2 | 0 | -0.2 | 0.2 |
|  | 2 | 66.4 | 13.2 | 20.3 | 0 | 0 | 0 |
| Tanzania | 1 | 72.2 | 12.7 | 15.1 | 0 | -5.1 | 5.1 |
|  | 2 | 71.4 | 7.6 | 21.0 | 0 | 0 | 0 |
| Uganda | 1 | 58.1 | 16.2 | 25.8 | 0 | 0 | 0 |
|  | 2 | 64.1 | 11.3 | 24.5 | 0 | 0 | 0 |
| Zimbabwe | 1 | 54.3 | 20.8 | 24.9 | 0 | 0 | 0 |
|  | 2 | 53.1 | 10.8 | 36.1 | 0 | 0 | 0 |

Abbreviations: pp, percentage points.

Notes: Primary analysis classified unknown antiretroviral therapy status among those aware as not on antiretroviral therapy; sensitivity analysis classified unknown status among those aware as on antiretroviral therapy. Change columns show sensitivity minus primary. Tanzania Round 1 had 4.5% missing antiretroviral therapy status, explaining the 5.1 pp shift from ‘diagnosed, not on antiretroviral therapy’ to ‘on antiretroviral therapy but not suppressed’ pathways.
